# The Impact of Expectation for Pain Relief on Orofacial Pain Treatment Outcomes

**DOI:** 10.3389/fpsyt.2021.734986

**Published:** 2021-11-02

**Authors:** Yaron Haviv, Sigal Mazor, Margolis Shani, Robert Yanko, Doron J. Aframian, Yehuda Zadik, Shiri Ben-David, Asaf Wilensky, Yair Sharav

**Affiliations:** ^1^Department of Oral Medicine, Sedation & Maxillofacial Imaging, Faculty of Dental Medicine, Hadassah Medical Center, Hebrew University of Jerusalem, Jerusalem, Israel; ^2^Department of Oral Medicine, Sedation & Maxillofacial Imaging, Faculty of Community Dentistry, Hadassah Medical Center, Hebrew University of Jerusalem, Jerusalem, Israel; ^3^Hebrew University Hadassah Medical School, Jerusalem, Israel; ^4^Department of Military Medicine, Faculty of Medicine, Hebrew University of Jerusalem, Jerusalem, Israel; ^5^Neuropsychiatric Clinic, Department of Psychology, Hadassah Medical Center, Hebrew University, Jerusalem, Israel; ^6^Department of Periodontology, Faculty of Dental Medicine, Hadassah Medical Center, Hebrew University of Jerusalem, Jerusalem, Israel

**Keywords:** chronic orofacial pain (OFP), expectations, recalled expectations, cognitive dissonance, temporomandibular disorder, desire

## Abstract

**Aims:** To examine the effects of expectations for pain relief on the objective and subjective outcome of chronic orofacial pain (OFP) treatment.

**Materials and Methods:** Sixty individuals referred to the Orofacial Pain Clinic at the Hebrew University-Hadassah School of Dental Medicine between 2015 and 2017 with OFP reported their expectation for pain relief upon initial consultation. They were also interviewed by telephone *after* treatment and asked to recall their expectations, referred to as “recalled expectations” (RE). Correlations between RE and treatment success were calculated from pain diaries, and from subjective pain improvement rates (PIR) reported by the patients.

**Results:** 21 males (35.0%) and 39 females (65%), mean age of 46.90 ± 15.77 years and mean pain duration of 49.07 ± 51.95 months participated in the study. All participants rated their expectations as “10” on a 0 to 10 scale during their first visit. RE did not correlate with diary ratings, (*P* = 0.773) but inversely correlated (−0.3) with PIR (*P* = 0.020) treatment outcomes.

**Conclusions:** Expectations for pain relief, reported as 10 on a 0–10 scale during the first appointment, may reflect the patient's *desire* for complete relief of their pain rather than their *expectations*. Clinicians should therefore be aware of the need for clear communication and wording when examining for expectations. Inverse correlation between recalled expectations and subjective outcome may be due to the nature of recalled expectations when patients already knew their treatment outcomes, and may be explained by the concept of cognitive dissonance.

## Introduction

Chronic orofacial pain (OFP) includes heterogeneous conditions, and treatment outcomes depend on various causes (1). Accumulating evidence suggests an association between patient pretreatment expectations and numerous health outcomes, and the predictive association between expectations and clinical outcomes. In a recent large multi-center study (2) chronic pain patients' expectations regarding pain relief and improvements in quality of life and functioning were measured *before the first visit* to the pain center. Higher expectations from treatment predicted reduced pain intensity, depressive symptoms, pain interference, and tendency to catastrophize, as well as satisfaction with pain treatment and global impressions of change at 6-month follow-up (2).

Despite the assumption that expectations from treatment would positively correlate with better treatment outcomes, patient expectation fulfillment was found to be unrelated to pain improvement following hip or knee surgery, when surveyed within 12 months *after* surgery, probably due to unrealistic expectations (3).

Yet, expectations from treatment as predictive of treatment outcome for OFP has not been investigated so far.

In light of the conflicting evidence between expectations measured *before* treatment and expectations fulfillment *after* treatment, we decided to examine expectations for pain relief, recorded during the initial consultation for treatment of OFP and also ask the same patients to recall their original expectation assessment score while conducting a telephone interview performed *after* treatment. These recollections were referred to as “recalled expectations” (RE). We hypothesized that higher expectations would correlate with greater pain relief in both instances; before and after treatment (4).

## Methods

The study was approved by the Ethical Committee Hadassah Medical Center, request no. 0132-17-HMO. All data were fully anonymized, Inform consents were waived according to Ethical Committees' instructions.

The medical records of OFP patients meeting our inclusion criteria attending the Orofacial pain clinic, at the Hebrew University-Hadassah School of Dental Medicine, between 2015 and 2017 were reviewed. Inclusion criteria were: over 18 years of age; diagnosis of primary chronic OFP for at least 3 months (1); at least one follow-up visit; available for telephone interview 3–6 months after initial consultation. During the first appointment a thorough history including pain intensity as well as expectation for treatment success marked on a 0 to 10 scale, demographic data, pain intensity and quality rated on a 0 to 10 verbal pain scale (VPS) over the previous week were recorded. Patients were asked if pain wakes from sleep, if the pain comes and goes in “attacks,” is constant or both i.e., constant pain with episodes of exacerbation. Pain distribution was charted by marking five areas on each side of the face; the total “number of surfaces” (NOS), represents pain spread, with maximum score 10 (5). Sleep quality and health-related quality of life (HRQoL) over the last month on a 0–10 numeric scale were recorded (6). For recording recalled expectations (RE) a structured telephone interviews using the expectation rate questionnaire were conducted by the same investigator (MS). The expectation rate questionnaire included six questions scored on a 0 to 10 scale and based on previously validated questionnaires (7, 8) (Appendix). Patients were asked to recall their original assessment of expected pain relief and then asked to assess their actual subjective pain improvement rate (PIR) on a scale of 0 to 10. Patients also submitted pain diaries in which daily pain intensity scores on a 0 to 10 scale were recorded daily.

### Statistical Methods

Significant differences in means were calculated using Student *t*-test or ANOVA. Correlations between two nominal variables were assessed using Chi square test. Pearson's Correlation between two continuous variables was calculated. Statistical tests were two-sided using a 5% significance level.

## Results

Out of 200 records, 100 met study criteria, and 60 patients were available for the telephone interview which was taken 3 to 6 months after last meeting and 21 to 24 months after first meeting. During the first visit the expectation rates by all patients were marked as 10. We suspect that these numbers reflect a desire for pain relief rather than expectation rates; to be discussed in detail below. These data were therefore discarded from further analysis. All analysis was then carried out only on the recalled expectations data (RE). Demographics, pain characteristics and RE are presented in Tables 1, 2. Phone questionnaire outcomes in relation to RE are presented in Table 3.

**Table 1:** Demographics, pain characteristics and recalled expectation rates (RE)^*^.

			N*	RE**	***P* value**
Gender		M	21 (35%)	7.95, 2.08	0.720
		F	39 (65%)	7.72, 2.62	
Associated pain related diseases		Healthy	22 (38.6%)	7.45, 2.59	0.860
		Pain related diseases	10 (17.5%)	7.90, 2.92	
		Other	25 (43.6%)	8.00, 2.24	
Laterality		Unilateral	33 (57.9%)	8.21, 2.38	**0.046**
		Bilateral	24 (42.1%)	7.08, 2.44	
Temporal pain characteristics		Attacks	17 (29.3%)	8.12, 2.59	0.740
		Constant	25 (43.1%)	7.52, 2.55	
		Both	16 (27.6%)	7.75, 2.23	
Pain wake from sleep		No	35 (60.3%)	7.60, 2.56	0.240
		Yes	23 (39.7%)	8.35, 2.03	
Pain relief		<50%	36 (70.6%)	7.59, 0.49	0.773
		≥50 %	15 (29.4%)	7.93, 0.54	
Quality	Burning	Yes	12 (20.7%)	6.08, 3.39	**0.007**
		No	46 (79.3%)	8.2, 1.96	
	Electrical	Yes	7 (12.1%)	9, 1.29	0.154
		No	51 (87.9%)	7.59, 2.53	
	Stabbing	Yes	18 (31%)	8.17, 1.85	0.399
		No	40 (69%)	7.58, 2.67	
	Throbbing	Yes	18 (31%)	8.0, 2.44	0.620
		No	40 (69%)	7.65, 2.47	
	Pressure	Yes	36 (62.1%)	7.58, 2.30	0.490
		No	22 (37.9%)	8.05, 2.70	
Diagnosis		Migraine or NVOP	21 (35%)	7.67, 2.44	0.387
		TMD	16 (26.7%)	8.06, 2.32	
		PTTN	10 (16.7%)	7.00, 3.20	
		TN	5 (8.3%)	9.60, 0.89	
		Other	8 (13.3%)	7.50, 20	
Medication type		AED	40 (85.1%)	8.05, 2.297	0.605
		TCA	3 (6.3%)	7.33, 0.58	
		SNRI	2 (4.2%)	6.00, 5.66	
		Abortive	2 (4.2%)	7.00, 2.83	

*Absolute numbers and percentages can be smaller than the cohort if answers were missing or ambiguous.

**RE- Recalled expectation rates inquired retrospectively during telephone interview rated from 0 to 10.

*NVOP, neurovascular orofacial pain; TMD, temporomandibular disorders; PTTN, post traumatic trigeminal neuropathic pain; TN, trigeminal neuralgia; AED, antiepileptic drugs; TCA, tricyclic antidepressants; SNRI, serotonin norepinephrine inhibitors; Abortive = Mostly NSAIDs. Others - include Atypical, burning mouth syndrome (BMS) and persistent idiopathic facial pain (PIFP)*.

**Table 2:** Demographic and pain characteristics in relation to RE^*^.

	N*	**Mean**	**Pearson correlation**	***P* value**
RE**	60	7.8, 2.42	−	−
Age	60	46.9, 15.77	0.073	0.58
Onset of pain (m)	60	49.07, 51.95	−0.185	0.16
NOS***	60	2.55, 1.77	−0.222	0.09
Sleep quality	47	6.45, 2.79	−0.081	0.59
HRQoL	43	6.12, 2.42	−0.121	0.44
Muscle tenderness score	56	2.80, 2.99	−0.083	0.54
VPS at intake	57	7.77, 1.79	0.087	0.54
VPS at last visit	20	6.30, 3.34	0.425	0.06
Pain improvement rate (PIR) (0 → 10)	60	4.68, 3.28	**−0.3**	**0.02**

*Absolute numbers and percentages can be smaller than the cohort if answers were missing or ambiguous.

**RE- Recalled expectation rates inquired retrospectively during telephone interview rated from 0 to 10.

***NOS, number of surfaces.

**Table 3:** Phone questionnaire outcome in relation to RE^*^.

	**Questions**	N* (%)	RE**	***P* value**
RE**	0 to 10 scale	60 (100%)	7.80 ± 2.42	-
Continued medical treatment	Yes	47 (79.7%)	9 ± 2.38	0.23
	No	4 (6.8%)	8 ± 2.45	
	Sometimes	8 (13.6%)	6.5 ± 2.67	
Discontinuation of treatment	Didn't help	20 (57.1%)	8.65 ± 2.16	0.20
	Pain stopped	3 (8.6%)	7.67 ± 2.51	
	Side effects or replaced with other treatment	6 (17.1%)	8.67 ± 2.16	
	Others	6 (17.1)	6.5 ± 3.15	
Connection between pain improvement and primary expectations	Yes, to a great extent	9 (15%)	8.44 ± 1.24	0.83
	Yes, to a small extent	5 (8.3%)	7.8 ± 2.49	
	Maybe there is a connection	12 (20%)	7.92 ± 2.91	
	No connection	34 (56.7%)	7.59 ± 2.43	
RE is influenced by***	clinic's reputation	16 (26.7%)	8.88 ± 1.45	6
	Attitude to the complaints	11 (18.3%)	8.45 ± 1.75	
	approach of the clinic workforce	11 (18.3%)	6.73 ± 2.68	
	other non-specific factors	22 (36.7%)	7.23 ± 2.84	

*Absolute numbers and percentages can be smaller than the cohort if answers were missing or ambiguous.

**RE- Recalled expectation rates inquired retrospectively during telephone interview rated from 0 to 10.

***Patient point of view.

A treatment outcome of at least 50% pain reduction on pain diaries did not correlate with RE [7.59 ± 0.49 (<50%) vs. 7.93 ± 0.539 (≥50%); *p* = 0.77]. However, patient subjective pain improvement rate (PIR) (4.68 ± 3.28) was inversely correlated with RE [Pearson's Correlation (−0.3); *p* = 0.02]. RE was not associated with gender, pain characteristics, diagnosis, or medications used (Table 1), nor with age, onset of pain, NOS, sleep quality, HRQoL, muscle tenderness score and VPS during initial consultation or at interview (Table 2).

## Discussion

Treatment outcomes, according to diary ratings, did not correlate with RE (*P* = 0.773). However, the subjective pain improvement rate (PIR) according to the patient's opinion of success was inversely correlated (−0.3) with RE (*P* = 0.020). These findings are puzzling, considering the established positive relationship between high expectations and treatment success (7). However, unlike previous studies that recorded expectations before treatment (7, 8), our study assessed recollection of expectations after treatment.

The subjective assessment of treatment outcome may have influenced the retrospective assessment of expectations. To note, the more objective measure for treatment outcome, pain diaries, was unrelated to recalled expectations. Yet recalled expectations did inversely correlate with the subjective assessment of pain improvement. One should be aware of the inherited differences between diary reporting and retrospective recollection, as patients tend to exaggerate recalled memory (9). Apparently, the more disappointed patients were from treatment outcome; the higher they assessed their pre-treatment expectations. This paradoxical finding might be explained by the concept of cognitive dissonance. Cognitive dissonance mechanisms are used in order to resolve the stress caused by the inconsistency experienced by a person who holds two or more contradictory beliefs, ideas or values, in order to reduce discomfort (10). In our study, patients who subjectively evaluated treatment outcome as poor may have experienced a cognitive dissonance as they had invested time and money as well high expectations, still subjectively assessed a poor outcome. In an attempt to reduce their disappointment and discomfort from the unsuccessful treatment compared to the effort invested, they exaggerated their pre-treatment expectations, legitimizing their invested efforts in the treatment because they expected much better results and anticipated their efforts would “pay off.” On the other hand, patients that evaluated their pain relief as adequate did not experience a dissonance; they sought treatment for pain and got better, and therefore adjusted their recalled expectations as being modest, meeting the real achievements. Nevertheless, it is also possible that the differences in expectation fulfillment may be due to unrealistic expectations (3). Interestingly, patients were unaware of the connection between pain improvement and their expectation rating. Expectations are created and sustained by a cognitive process. An event, however, can be desired but not expected, (11). Therefore, expectations can also be expressed as desires, wishes and hopes (12).

During the first visit all patients had a maximum expectation of 10. The fact that the answer for the primary “expectations” question was 10 out of 10 for all patients, even though most of them experience long lasting resistant pain that lasted months to years is unrealistic and doesn't make any sense. Therefore, we suspect that ratings were an expression of desire for pain relief (DPR) rather than a genuine expectation. Thus, we assume that an individual's *desire* for relief may contribute to subsequent pain reduction, responding to the need to experience a treatment as effective. The effect of the DPR on analgesia is represented by the idea that greater threats may lead to a greater need for relief. However, expectancy but not DPR contributed to the magnitude of placebo analgesia in participants undergoing experimentally-induced pain (13). As mentioned above, we therefore suspect that the high “expectation,” expressed by our patients during their initial appointment, were in fact a measure of their “desire” for pain relief and consequently had a minimal effect on treatment outcome.

We most probably failed to explain to our patients more explicitly the difference between expectation and desire for pain relief. It seems advisable therefore to be more careful and clearly communicate when asking about patient expectations (14).

To conclude, this is a preliminary study that points to the need for careful communication and choice of correct wording when examining expectations for treatment outcome. One should be aware of unrealistic “expectations” when in effect they are more an expression of a desire for pain relief.

## Data Availability Statement

The original contributions presented in the study are included in the article/supplementary material, further inquiries can be directed to the corresponding author.

## Ethics Statement

The studies involving human participants were reviewed and approved by Ethical Committee Hadassah Medical Center, request no. 0132-17-HMO. Written informed consent for participation was not required for this study in accordance with the national legislation and the institutional requirements.

## Author Contributions

YH, YS, and SM made substantial contributions to the study conception and design, acquisition of data, and analysis and interpretation of data. SB-D, DA, and YZ drafted the manuscript and provided critical psychological interpretation. AW, YH, RY, YS, and SB-D wrote, revised, and approved the manuscript. SM analyzed the data and performed the statistical analysis, revised, and approved the manuscript. All authors contributed to the article and approved the submitted version.

## Conflict of Interest

The authors declare that the research was conducted in the absence of any commercial or financial relationships that could be construed as a potential conflict of interest.

## Publisher's Note

All claims expressed in this article are solely those of the authors and do not necessarily represent those of their affiliated organizations, or those of the publisher, the editors and the reviewers. Any product that may be evaluated in this article, or claim that may be made by its manufacturer, is not guaranteed or endorsed by the publisher.

## Appendix

____________________________________________________

1. What were your expectations, in the first meeting, for the treatment success, on a 1–10 scale? ______

2. To what extent did your condition improve following the treatment you received at the pain clinic, on a 1–10 scale? (Improvement rate) ______

3. Have you persisted in the treatment you received?

A. Yes

B. No

C. sometimes

4. Have you stopped treatment? If so, why?

A. The treatment did not help

B. The pain passed

C. Side Effects

D. Treatment has been replaced

G. Other

E. I am still in therapy

5. Do you think there is a connection between the level of expectations you had when you reached the clinic and the success of the treatment?

A. Yes, to a great extent

B. Yes, to a small extent

C. Maybe there is a connection

D. There is no connection

6. What affected your level of expectations the first time you got to the clinic?

A. Clinic reputation

B. Respectful attitude to my complaints

C. Attitude of the treating staff

D. other, specify__________________________(*open question*)
